# Wireless system for recording evoked potentials

**DOI:** 10.1186/s12576-024-00923-6

**Published:** 2024-05-21

**Authors:** Yutaro Oguma, Toshi Nakajima, Megan Elizabeth Young, Ryoi Tamura

**Affiliations:** 1 Department of Integrative Neuroscience, Graduate School of Medicine and Pharmaceutical Sciences, University of Toyama, Toyama, Japan; 2 School of Medicine, University of Toyama, Toyama, Japan; 3 Department of Physiology, Faculty of Medicine, Kindai University, Osaka, Japan

**Keywords:** Wireless, Evoked potential, Recording and stimulation system, Custom-made

## Abstract

Experiments measuring evoked potentials require flexible and rapid adjustment of stimulation and recording parameters. In this study, we have developed a recording system and an associated Android application that allow making such adjustments wirelessly. The system consists of 3 units: for stimulation, recording and control. Most of the modules in this system are custom made, although the stimulator and tablet are off-the-shelf products. When installed on the tablet, our Android application allows wireless communication with the control unit from a distance of 5 m. In testing, the recording unit had low internal noise and displayed signals faithfully. Upon receiving commands from the control unit, the stimulation unit produced precisely timed pulse outputs. Using this system, we were able to record evoked field potentials in the dentate gyrus of a rat; responses increased as expected with increasing stimulation pulse amplitude and duration.

## Background

Recording biologically relevant potentials evoked by electrical stimulation is a fundamental electrophysiological technique with broad applications not only in basic medical research, but also in clinical and educational settings [1]. It often requires flexible adjustment of various parameters, such as stimulus intensity, latency, duration, and frequency, depending on the purpose of the experiment [2]. However, when using conventional general purpose electrical stimulators, such operations are usually performed manually and are therefore time-consuming. Although integrated systems consisting of stimulation, recording, and control units are commercially available, such systems are often costly, making it desirable to build a system with the same capacity using more readily-available components.

To achieve good performance, such a system must overcome electrical noise, limitation of laboratory space, and the complexity of connections among its modules (devices). To obtain high quality signals with minimal noise, it is desirable to place both a stimulator (with an isolator) and an amplifier near the subject, with the control unit (e.g., a PC) at a distance. However, the use of high-performance PCs, which tend to generate larger electrical noise, limits the placement of the devices in the experimental space, reducing the flexibility of the system installation. Moreover, the wiring becomes longer and more complex as the number of interconnected devices increases; this makes it difficult to check for correct wiring and ground loops, and to add new devices with cables. One effective way to avoid these problems is to use wireless remote control.

In this study, we developed an integrated system that allows flexible configuration of stimulation parameters and monitoring of evoked potentials via Bluetooth communication with a tablet. We then evaluated its usability by recording evoked potentials from the dentate gyrus (DG) of the rat. Some results of this study have already been reported at the 69th Annual Meeting of the Chubu Japan Physiological Society.

## Methods

### System specifications

**Fig. 1:**
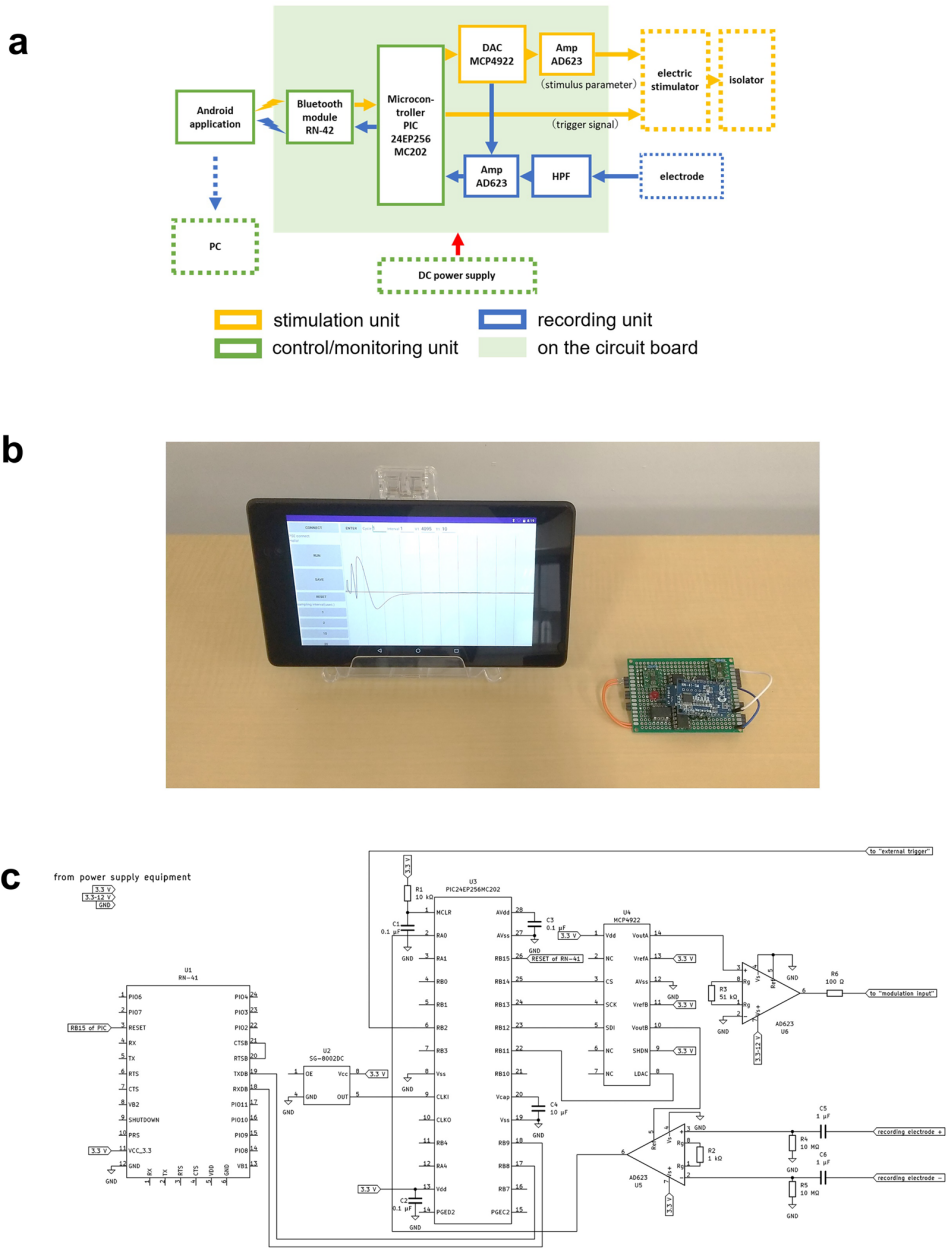
System configuration. **a** Overall block diagram. Each module, either hand-made (solid border) or standard product (dotted border) is color coded according to which unit it belongs to. Shaded area indicates the hand-made circuit board (device). Arrows show command/signal flow. Amp; amplifier, DAC; digital to analog convertor, HPF; high-pass filter. **b** Photograph showing the device and tablet with a running application. **c** Schematic of the circuit in the shaded area in (**a**)

The present system was broadly divided into three units: (1) stimulation, (2) recording, and (3) control/monitoring (Fig. 1a). The general-purpose electrical stimulator and the tablet used their own power supply, while the rest of the system (instrumentation amplifiers, microcontroller, Bluetooth unit, etc.) was supplied with 3.3 V from a stabilized power supply (KA-064, Kyoritsu Electronics Industry, Japan).

#### Stimulation unit

The stimulation unit consisted of a stimulator (SEN-3201, Nihon Kohden, Japan), isolator (SS-201 J, Nihon Kohden, Japan), buffer amplifier (AD623, Analog Devices, Inc., USA, gain × 3) and digital to analog converter (DAC, MCP4922, Microchip Technology Inc., USA, 12 bit, 10 kHz) (Fig. 1a, c). The PIC microcontroller (PIC24EP256MC202, Microchip Technology Inc., USA) sent the trigger signal directly to the stimulator for stimulus generation (Fig. 1a); it also sent output control commands to the DAC. Based on this command, the DAC generated a pulse wave with any specified stimulation parameters (duration and amplitude) and sent it to the modulation pin of the stimulator via the buffer amplifier (Fig. 1a); we placed this buffer amplifier to match the DAC output range (0–3.3 V) with the range of the modulation input (0–10.0 V). Although various combinations of stimulator and isolator settings are possible, in this study, we set the stimulator to produce a positive square-pulse of 2 ms fixed duration with its maximum output (ca. 50 V). Then, the stimulator output a pulse with arbitrary duration of up to 2 ms receiving the command from the DAC. The isolator was set to current mode.

#### Recording unit

The recording unit was a single-channel amplifier with a simple high-pass CR filter consisting of a surface-mount capacitor and resistor to remove the DC component of the signal (Fig. 1a, c; cutoff frequency 0.017 Hz). To reduce circuit complexity and obtain good common-mode rejection performance, a single-chip instrumentation amplifier (Analog Devices, AD623) was used; the gain was set to 100x (Fig. 1a, c). The bandwidth is about 10 kHz. This gain was chosen as the optimal gain considering the maximum amplitude of the DG field potentials measured (10–15 mV), the dynamic range of the ADC on the PIC (12 bit, 0–3.3 V), and the frequency characteristics of the AD623. Since the ADC on the PIC works only on positive voltages, the output of the amplifier was shifted by + 1.6 V (Fig. 1c).

#### Control and monitoring unit

The control and monitoring system consisted of a tablet equipped with Android OS (Nexus 7, Google, USA, Android version 6.0.1), a PIC microcontroller, and a Bluetooth unit (RN-41, Roving Networks, USA). We chose Bluetooth as a means of wireless operation because (1) it has sufficient range (10 m) for most experimental setups, (2) it is built into most tablets and smartphones and can be easily controlled from the Android application layer, (3) small, low-cost modules suitable for convenient implementation on the PIC side are readily available, and (4) with low power consumption, it produces minimal electrical noise that could obscure target biological signals.

**Fig. 2:**
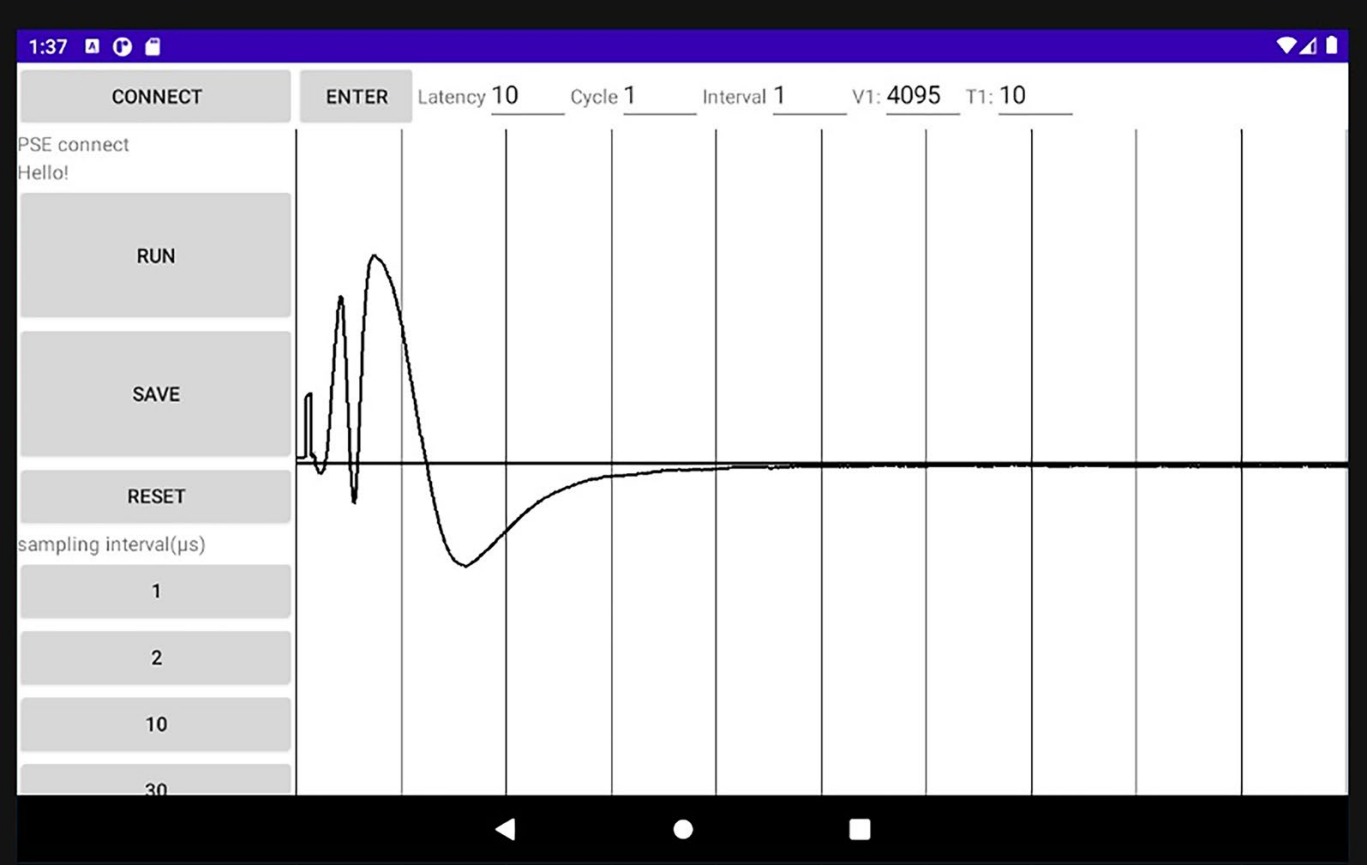
Display screen on tablet, while the application is running. Buttons (gray shaded rectangles, left) and text fields (top) allow users to control the system. Time course of recorded evoked potential is displayed to the right of the buttons

The tablet was the interface that allowed an experimenter to specify the stimulation parameters and to display the waveforms of the evoked potentials. The tablet application was developed in Java using Android Studio (Google, USA) and the PIC firmware was written in C using MPLAB® X Integrated Development Environment (Microchip Technology Inc., USA). Figure 2 shows an example display of the tablet. The experimenter entered the basic stimulation parameters, i.e., amplitude (12 bits), latency and duration of the pulse (0.1 ms increments for each), stimulus interval (1 s increments), and number of stimuli, into the text fields at the top. In addition, the buttons at the bottom left of the display allowed the experimenter to select a sampling interval (1, 2, 10, 30 or 50 µs). Upon hitting the “ENTER” button, the parameter values were sent to the PIC on the circuit board via Bluetooth (Fig. 1a).

Tapping the “RUN” button initiated stimulation and recording. For stimulation, the PIC sent the DAC the amplitude value, followed by the specified latency period. Then, the PIC sent the latch signal to commence the stimulation, causing the output voltage to rise. After the specified pulse duration, another latch command was sent to drop the output voltage to 0 V. For recording, an appropriate phase-locked loop (PLL) of the PIC timer was determined based on the selected sampling interval, and AD conversion of the input signal from the amplifier (12 bit resolution) was started. After 2000 sampling cycles, the AD conversion was stopped, after which recorded data was sent to the tablet via Bluetooth, and the waveform was displayed on the tablet. Then, a temporary data file was created in a hidden folder. Tapping the “SAVE” button saved the file as a csv format, whereas the “RESET” button deleted the temporary data file. This series of processes was automatically repeated for the number of trials at preset trial intervals. In this study, we used direct memory access for AD conversion to save the CPU time.

### Evaluation of fundamental performance

To investigate the basic characteristics of the system, we tested four key parameters in an experimental room with electromagnetic shielding. First, we recorded internal noise (voltage variations recorded with both differential inputs connected to ground). Second, we measured the level of recorded waveform distortion after a continuous square wave of 10 mV amplitude, 20 ms period, and 50% duty cycle was input from the stimulator to the recording unit. Third, we tested the reliability of wireless communication by increasing the distance between the tablet and the device up to 5 m with the same input as above and checking for operational errors. Finally, the input–output linearity was tested. To measure output current, we connected a 1 kΩ resistor to the output terminals of the isolator and sent a pulse while monitoring the difference in voltage between both sides of the resistor with an oscilloscope. The current was then calculated using Ohm’s law. The stimulus intensity was set with the tablet device. The DA output setting value was gradually increased from 0 to 4000 in 250 steps.

### Physiological assessment: evoked potential recording in the rat DG

As in vivo recording of field potentials triggered by perforant pathway stimulation in the DG of the rat is a well-established and widely used technique in electrophysiological research, we chose it as a relevant paradigm for verifying the performance of our system.

In brief, when the perforant pathway is stimulated, the granular cell layer of the DG produces a relatively high-amplitude, characteristic waveform consisting of a population excitatory postsynaptic potential (pEPSP) and a population spike (PS) [3–8].

We recorded in the DG of one (naïve 350 g male) Sprague–Dawley rat. All animal care and experimental procedures were performed with the approval of the University of Toyama Animal Care and Use Committee (approval #: A2022MED-13).

Detailed methods for performing field potential recordings in the DG can be found in previous literature; for examples, see [3–8]. Briefly, the rat was administered medetomidine (0.02 mg/kg, i.m.) and midazolam (0.3 mg/kg, i.m.) for induction anesthesia; the anesthesia was maintained with sodium pentobarbital (10 mg/kg, i.p.). The head was mounted on a stereotaxic apparatus, and the skull was exposed. Two stainless steel screws were fixed into the temporal bone, one on each side, and a lead wire was soldered to each one. Then, two holes were made in the right temporal bone above the stimulation and recording sites (perforant pathway and DG, respectively) based on the rat brain atlas [9]. The stimulation electrode (polyurethane-coated concentric stainless steel electrode) and recording electrode (polyurethane-covered monopolar stainless steel electrode) were slowly advanced through these holes until they reached the target sites, whereupon stimulation and recording were initiated. The positions of both electrodes were fine-tuned to obtain optimal responses based on the observed evoked field potential waveforms (the stimulation, at − 7.8 mm anterior, 4.4 mm lateral, 5.0 mm ventral from bregma; the recording, at − 4.0 mm anterior, 2.5 mm lateral, 3.7 mm ventral from bregma).

The tablet was placed about 3 m from the rest of the system and the rat. Since the magnitude of the evoked response depends on both stimulus intensity and duration, we performed two sets of recordings, incrementing the current value and duration in turn. First, stimulus duration was fixed at 1.0 ms, while the current value was varied in 18 steps from 4 to 220 µA (step sizes were as follows: 4 μA step between 4 and 20 μA, 8 μA step between 20 and 60 μA, and 20 μA step between 80 and 220 μA). As 60 µA was the lowest intensity at which a significant PS was observed, current value was then fixed at 60 µA, while the stimulus duration was varied from 0.1 to 1.5 ms in 0.1 ms steps. For each set of parameters, five recordings were performed with 1 s between stimuli. These five were then averaged to calculate PS amplitude and pEPSP slope for each parameter pair.

## Results

### Fundamental performance

**Fig. 3:**
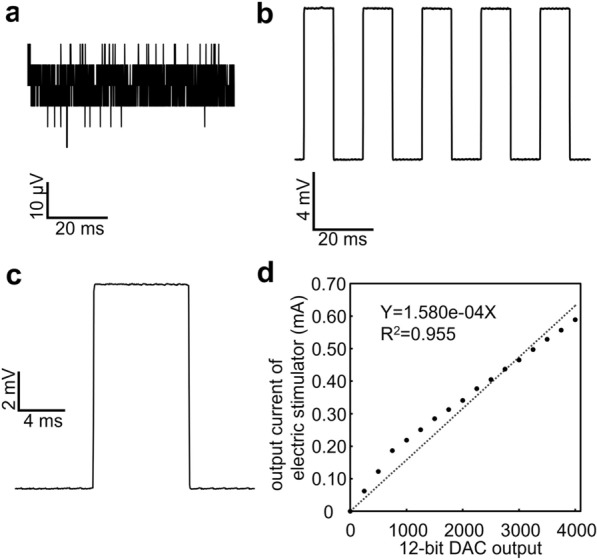
Fundamental performance. **a** Internal noise recorded with the input pins connected to ground. **b** Recorded square waves. **c** Magnified waveform of the square wave shown in b. **d** Relationship between the current value set with the tablet and amplitude of the recorded waveform with the dashed regression line obtained by means of a linear regression (Y = 1.580e-04X, R^2^ = 0.955)

We found that our system was able to replicate generated stimuli with high fidelity, avoiding nonlinear changes in amplitude and signal distortion. The internal noise observed when the input terminal of the recording section was grounded was ~ 40 µV p–p (Fig. 3a). The recorded waveform obtained by inputting a square pulse train generated by the stimulator was sharp on both the rising and falling edges, and there were no apparent transient responses, such as ringing, overshooting, differential waveform conversion, or integral waveform conversion (Fig. 3b, c). We repeated the monitoring operations (driving the stimulator and recording its output) several times every 1 m up to 5 m in the experimental room. Even at the maximum distance (5 m), there were no communication errors between the tablet and the device. As shown in Fig. 3d, the relationship between the stimulation parameters set on the tablet and the recorded waveform amplitude was approximately linear, although the waveform amplitudes tended to shift upward in the lower setting range.

### Evoked potential recording in the rat DG

**Fig. 4:**
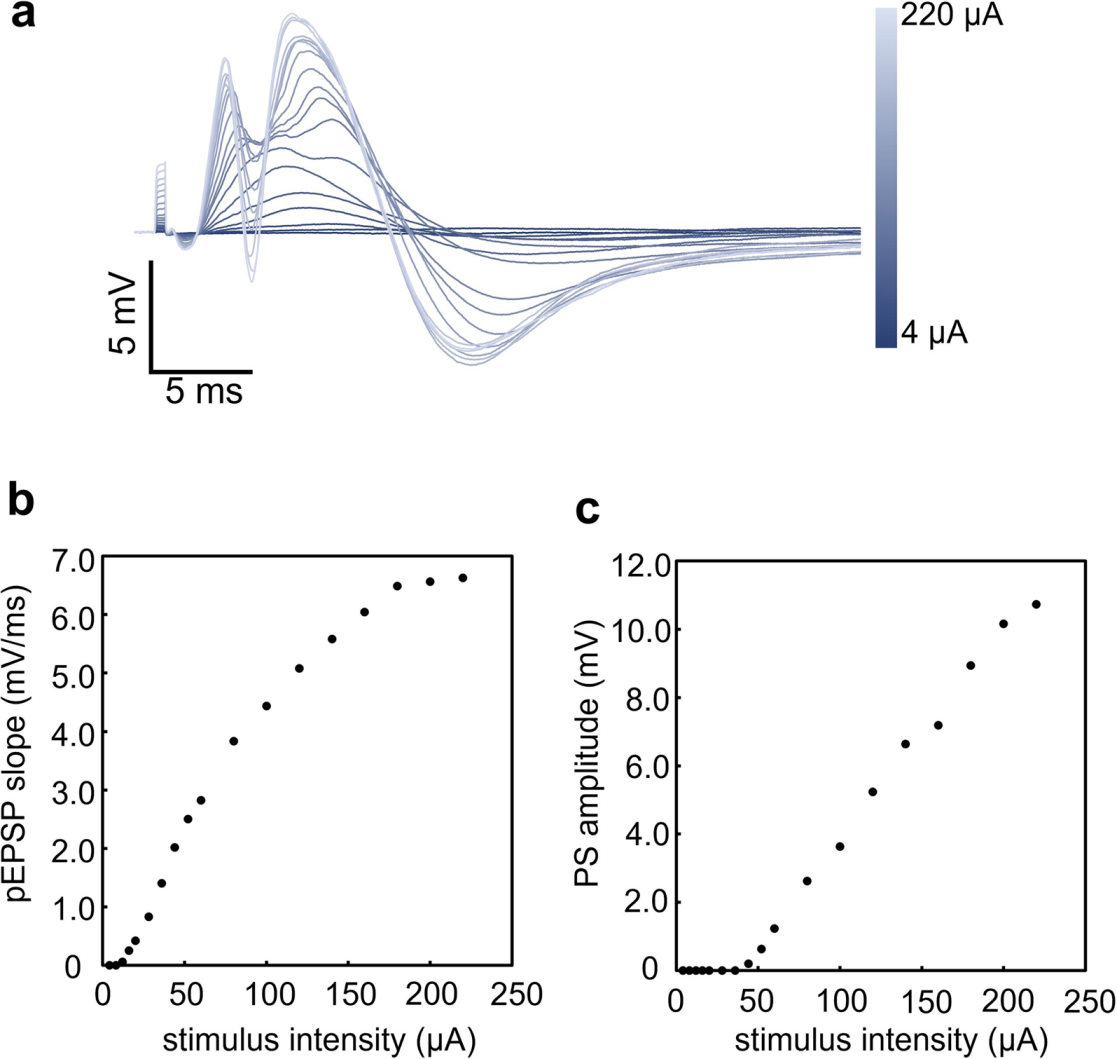
Current-increment experiment. **a** Superimposed waveforms of evoked potentials with current intensity varying from 4 to 220 µA, as indicated with the gradient bar on the right. **b**, **c** pEPSP slope (ordinate in **b**) or PS amplitude (ordinate in **c**) as a function of current intensity

By stimulating the perforant pathway, we successfully recorded evoked field potentials in the rat DG (pEPSP and PS). When input current was incremented from 4 to 220 µA with a fixed pulse duration of 1 ms, recorded field potentials were augmented as expected, with both pEPSP slope and PS amplitude increasing as a function of current intensity (Fig. 4a–c). The pEPSP appeared at 12 μA (pEPSP slope, 0.06 mV/ms), enhanced in proportion to current intensity, and reached 6.63 mV/ms in slope when the current was maximum (220 μA). PS appeared at 44 μA (0.2 mV in amplitude), which was later than pEPSP. It likewise increased in proportion to current intensity and plateaued (10.7 mV at 220 μA).

**Fig. 5:**
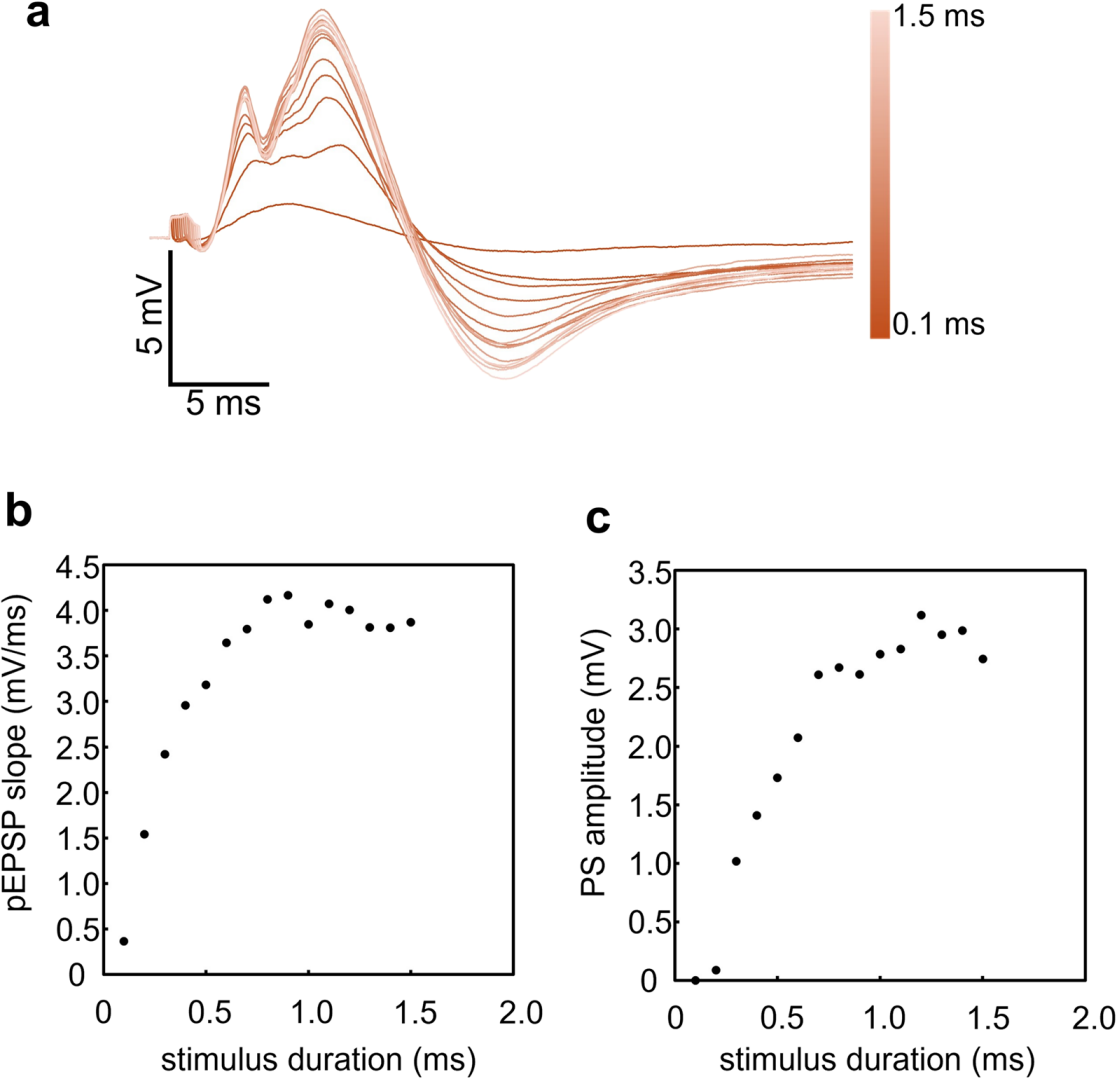
Duration-increment experiment. **a** Superimposed waveforms of evoked potentials with stimulus duration varying from 0.1 ms to 1.5 ms. **b**, **c** pEPSP slope (ordinate in **b**) or PS amplitude (ordinate in **c**) as a function of stimulus duration

Figure 5a shows the waveforms recorded while holding the stimulus current at 60 µA and incrementing the duration from 0.1 to 1.5 ms. As stimulus duration increased, changes qualitatively similar to those recorded in the current-increment experiment were observed. Specifically, a pEPSP appeared when the duration was 0.1 ms (pEPSP slope, 0.37 mV/ms), increased in slope as the duration increased, and the slope reached 4.17 mV/ms when the duration was 0.9 ms. A PS (0.09 mV) appeared when the duration was 0.2 ms and plateaued (3.12 mV) at 1.2 ms stimulus duration (Fig. 5a–c).

## Discussion

### Fundamental performance

The internal noise of the device was ~ 40 µV p–p. This is sufficiently low for relatively large-amplitude electrophysiological measures such as hippocampal evoked potentials, evoked electromyograms such as M and F waves, and slow waves during sleep to be detected with high fidelity. Of course, this noise level can be problematic for smaller biological measures such as fast EEG waves (beta, gamma, and ripple) using the present system. However, these smaller waves can be reasonably recorded by increasing the amplifier gain, although the higher gain setting results in a narrower bandwidth.

The system faithfully reproduced the input generated by the stimulator without apparent distortion and transients. The amplitude linearity between the stimulation set with the tablet and the recorded waveform was largely maintained, suggesting that detailed amplitude adjustment is not necessary in our system. In addition, the system performed reliably even when the tablet and the circuit device were separated by 5 m. Thus, with our system one can easily make wireless connections between experimental devices, reducing the number of wires, especially long cable connections, making it possible to avoid various electrical problems such as connection and contact failures, ground loops, short circuits, and electrical noise.

### Evoked potential recording in the rat DG

With the present system, typical evoked potentials (pEPSP and PS) were observed in the DG in response to perforant pathway stimulation. In the current-increment experiment, the pEPSP appeared and gradually increased its amplitude, followed by the PS, which also gradually increased its amplitude. In the duration-increment study, a similar result to that of the current-increment experiment was observed. These results are consistent with the basic physiological concept of the current–duration relationship [10]. We therefore confirmed that, with a simple manipulation on the tablet, one can set the stimulation parameters flexibly and change them quickly in reference to a displayed waveform. The results in the in vivo experiments were obtained, while the tablet was at a fixed distance (3 m) from the animal; however, we found that the tablet could be operated from anywhere in our laboratory space (3.8 × 6.7 m).

Minor adjustments to our Android application could enhance our current system by enabling the presetting of stimulation parameters using a configuration file. This would be particularly useful for experiments that involve iterating the same stimulation-recording sequence while varying the current intensity or stimulus duration. Furthermore, modifications to the PIC firmware would empower our system to generate stimulus trains with intricate temporal structures—prerequisites for the study of neural plasticity, such as paired-pulse facilitation and inhibition, post-tetanic, short- and long-term potentiation, and long-term depression.

We used the same preparation to perform the experiments for Figs. 4 and 5. However, the responsiveness to the stimulation was facilitated in the experiment shown in Fig. 5 (e.g., the responses to the stimulation of 1 ms duration with 60 µA); certain changes in stimulus–response efficacy may have occurred between the 2 experiments, such as changes in microenvironment around the electrode’s tip, sensitivity of neural tissue to stimulation, etc.

### Implications in other fields

Beyond the field of basic science research, the use of our present system extends to medical education and clinical examination. In laboratory exercises, students can use familiar machines (Android devices) to control stimuli and check results (recorded waveforms) through simple and easy operations. In addition, the wireless nature of the control system allows them to avoid problems such as disconnection and short circuits, which promotes educational efficiency. At the bedside, the system developed in this study will be of use in clinical settings that employ electrophysiological techniques, such as electroencephalography, electrocardiography, electromyography, deep brain stimulation, electrooculography and electroretinography, and auditory brainstem response. In this setting, the wireless operation of electrophysiological devices can facilitate zoning, such as separating clean from contaminated areas in operating rooms, promoting flexible collaboration between medical and technical professionals.

### Cost advantages

Our system offers advantages in terms of cost savings in research. There have indeed been several recording [11, 12] and stimulation-recording systems [13] featuring low-cost amplifiers. Compared with these systems, the major advantage of our system is the wireless, dual control of an amplifier and a conventional general purpose electrical stimulator. If a conventional stimulator, isolator, and an Android-based device (a tablet or smartphone) are already available, additional cost is only applicable to a custom-made control device. The control device is made up of commercially available parts (PIC, Bluetooth module, instrumentation amplifier, universal board, etc.) and the total cost is a few thousand yen, or several tens of US dollars. In sum, our system offers wireless stimulation and high-quality recording with low cost by combining existing general-purpose equipment with an inexpensive custom-made control device.

## Conclusion

Our system exhibits solid fundamental performance, providing a high signal to noise ratio suitable for various electrophysiological measures. The system reliably reproduces input signals without distortion, offering flexibility in setting stimulation parameters. Its wireless capabilities enhance practicality, allowing seamless operation within a laboratory space. The system's adaptability extends its utility to educational and clinical realms, providing a cost-effective solution for research, medical education, and clinical examinations. In summary, the wireless system combines efficiency, flexibility, and cost-effectiveness, making it a valuable tool for advancing research.

## Data Availability

The data sets used and/or analyzed during the current study are available from the corresponding author on reasonable request.

## Abbreviations

EEG: Electroencephalography
DAC: Digital to analog converter
DG: Dentate gyrus
PLL: Phase-locked loop
pEPSP: Population excitatory postsynaptic potential
PS: Population spike
